# Bedaquiline Targets the ε Subunit of Mycobacterial F-ATP Synthase

**DOI:** 10.1128/AAC.01291-16

**Published:** 2016-10-21

**Authors:** Subhashri Kundu, Goran Biukovic, Gerhard Grüber, Thomas Dick

**Affiliations:** a Department of Microbiology and Immunology, Yong Loo Lin School of Medicine, National University of Singapore, Singapore; b School of Biological Sciences, Nanyang Technological University, Singapore

## Abstract

The tuberculosis drug bedaquiline inhibits mycobacterial F-ATP synthase by binding to its c subunit. Using the purified ε subunit of the synthase and spectroscopy, we previously demonstrated that the drug interacts with this protein near its unique tryptophan residue. Here, we show that replacement of ε's tryptophan with alanine resulted in bedaquiline hypersusceptibility of the bacteria. Overexpression of the wild-type ε subunit caused resistance. These results suggest that the drug also targets the ε subunit.

## TEXT

The diarylquinoline bedaquiline (trade name Sirturo, code names TMC207 and R207910) is a new tuberculosis drug discovered by Koen Andries and colleagues (1, 2, 16). The drug was shown to inhibit F-ATP synthase of mycobacteria (1). Genetic, biochemical, and modeling studies demonstrated that the c subunit of the F-ATP synthase, forming the membrane-embedded rotary ring of the enzyme, contains the binding site of the drug (1–9). Recent structural studies suggest that bound bedaquiline prevents the rotor ring from acting as an ion shuttle and thus stalls the ATP synthase operation (9).

Using recombinant purified F-ATP synthase ε subunit, which plays a key role in coupling proton translocation across the membrane with the catalytic site events in the cytoplasm, we showed previously via tryptophan fluorescence and nuclear magnetic resonance (NMR) spectroscopy that the drug interacts with this subunit near its unique and Mycobacterium-specific N-terminal tryptophan residue 16 (10). Based on these *in vitro* data, we proposed that bedaquiline may target, in addition to the c subunit, the ε subunit of F-ATP synthase (10). Here, we carried out genetic studies to provide *in vivo* evidence for this second binding site of bedaquiline on the F-ATP synthase.

**FIG 1:**
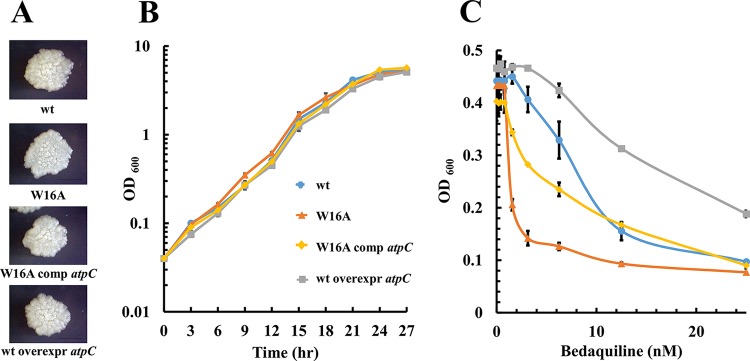
Growth and bedaquiline susceptibility of wild-type M. smegmatis and various genetic derivatives. (A) Growth on solid medium. Colonies are shown after 4 days of incubation. (B) Growth in liquid medium. (C) Bedaquiline growth inhibition dose-response curves. wt, wild-type M. smegmatis; W16A, M. smegmatis *atpC*^W16A^; W16A comp *atpC*, M. smegmatis *atpC*^W16A^ complemented with a wild-type copy of the gene encoding the ε subunit via the introduction of plasmid pMV262-*atpC*; wt overexpr *atpC*, wild-type M. smegmatis overexpressing wild-type ε protein via introduction of pMV262-*atpC*; OD_600_, optical density at 600 nm. The experiments were carried out three times independently. Mean values with standard deviations are shown.

To test our hypothesis that bedaquiline binds to the ε subunit near its tryptophan 16 residue, we replaced this amino acid with alanine by mutating the corresponding codon in the genome of Mycobacterium smegmatis mc^2^155 (ATCC 700084). We predicted that this amino acid exchange would change the bedaquiline susceptibility of the bacteria, either to hyposensitivity, if the drug binds more weakly to the mutated form of the ε subunit, or to hypersensitivity, if the mutation allows stronger binding. Site-directed, oligonucleotide-based genome mutagenesis (“recombineering” [11]) was carried out as described previously (12). Briefly, electrocompetent M. smegmatis harboring plasmid pJV53 and thus expressing the mycobacterial phage Che9c recombineering genes gp60 and gp61 was transformed with the double-stranded DNA oligonucleotide GGTGTGGCTGATCTGAACGTCGAGATCGTCGCCGTCGAGCGTGAGCTCGCGTCCGGACCCGCTACGTTCGTGTTCACCCGCACCACCGCCGGTGAGATCG (Integrated DNA Technologies, USA) containing the desired GCG alanine codon (underlined) to replace the genomic TGG tryptophan codon in position 16 of the ε subunit-encoding *atpC* gene. Colonies were PCR screened with 2 different primer pairs both containing the forward primer GCGCTTCCTGAGCCAGAACATGA. One pair contained the reverse primer CGAACGTAGCGGGTCCGGACCA, matching the wild-type codon for tryptophan, and one pair contained the reverse primer CGAACGTAGCGGGTCCGGACGC, matching the mutated codon for alanine. For colonies that showed positive PCRs with the pair containing the primer matching the mutated version and a negative PCR with the pair containing the wild-type version of the gene, the introduction of the desired mutation was verified by sequencing (AIT Biotech, Singapore). Figure 1A and B show that the resulting strain M. smegmatis *atpC*^W16A^ displayed a growth behavior on Middlebrook 7H10 agar and in 7H9 broth (Becton Dickinson, USA) that was indistinguishable from that of the parental wild-type strain. This shows that the tryptophan-to-alanine alteration in the ε subunit of the F-ATP synthase did not affect bacterial growth (13). To determine whether the amino acid replacement changed bedaquiline susceptibility of the bacterium, growth inhibition dose-response curves were determined using the broth dilution method as described earlier (14) and bedaquiline purchased from Genegobio (Los Angeles, CA). Figure 1C shows that whereas wild-type M. smegmatis showed a MIC_50_ of 10 nM, M. smegmatis *atpC*^W16A^ showed a MIC_50_ of 2 nM, a 5-fold-increased sensitivity to bedaquiline. The observed hypersusceptibility of the bacterium may suggest that the replacement of the bulky tryptophan with an alanine provides more space for a stronger binding of the drug to the ε subunit. The hypersensitivity phenotype was complemented by the introduction of a wild-type copy of *atpC* carried by the plasmid pMV262-*atpC* and expressed by the plasmid's *hsp60* promoter (Fig. 1C). Together, these results suggest that bedaquiline interacts with the ε subunit of the F-ATP synthase in intact bacilli. pMV262-*atpC* was constructed by inserting a PCR-amplified DNA fragment of the coding sequence of the *atpC* gene into plasmid pMV262 via its BamHI and PstI sites (15). As the *atpC* coding sequence contained an internal BamHI site, the cloning was carried out in a two-step PCR process to eliminate this restriction site by introduction of a silent mutation. First, two overlapping *atpC* fragments, lacking the original BamHI site, were amplified using the primer pairs Forward-BamHI (GGTGTGGATCCGCTGATCTGAACG)/Internal-reverse (CTCCACGAGGATTCGGACGGTCTC) and Internal-forward (GAGACCGTCCGAATCCTCGTGGAG)/Reverse-PstI (ATGGACGCTGCAGTCGATGTGACTAG). Then, using these two fragments as the template, the entire (BamHI site-mutated) *atpC* coding sequence was amplified with primers Forward-BamHI and Reverse-PstI.

To further confirm the binding of bedaquiline to the ε subunit *in vivo*, we carried out an overexpression experiment. If the drug indeed inhibits F-ATP synthase in intact bacteria by binding to the ε subunit of the enzyme, increasing the intracellular target concentration, i.e., overexpression of the wild-type ε subunit in wild-type bacteria, is expected to result in reduced sensitivity, i.e., increased resistance to bedaquiline. Figure 1C shows that this is the case. Wild-type M. smegmatis carrying wild-type ε subunit-overexpressing plasmid pMV262-*atpC* showed a 2.5-fold increase in MIC_50_, from 10 nM to 25 nM, compared to wild-type bacteria.

Taken together, these genetic results support a model in which bedaquiline inhibits mycobacterial F-ATP synthase via a novel second mechanism involving the enzyme's ε subunit in addition to binding to its c subunit. Our data suggest that the drug interacts with the ε subunit and that this binding occurs close to tryptophan 16 of the protein. The results further suggest that the replacement of the bulky side chain of tryptophan 16 with the smaller side chain of alanine allows for a tighter binding of the drug. In conclusion, we provide *in vivo* evidence for a second binding site of the new tuberculosis drug bedaquiline involving subunit ε of the mycobacterial F-ATP synthase. This information may be useful for the discovery of the next generation of diarylquinolines. In this context, it is important to note that in our previous biophysical *in vitro* and modeling studies (10), we proposed a detailed binding epitope involving in addition to tryptophan 16, characterized here genetically, arginine 37 and threonine 19 in the ε subunit and phenylalanine 50 in the c subunit. To begin to dissect the details of the second binding epitope of bedaquiline on the F-ATP synthase genetically, we replaced arginine 37 (CGG) in the ε subunit with glycine (GGG) by recombineering. We were able to generate viable bacteria carrying the desired mutation, and the mutated strain had the same generation time as that of wild-type bacteria, i.e., it showed wild-type-like growth behavior. Interestingly, bedaquiline MIC determinations showed no shift in drug susceptibility in the mutant M. smegmatis strain in which arginine 37 was replaced by glycine (data not shown). There are several possible explanations why changing arginine 37 in the ε subunit had no effect on bedaquiline MIC. First, the proposed binding model suggesting an interaction with arginine 37 is not correct and may require modification. Alternatively, the proposed binding model may be correct; however, the contribution of arginine 37 to bedaquiline binding may not be detectable by genetic means, i.e., the interaction might be too weak to cause phenotypic consequences when interrupted. In general terms, genetic confirmation of a biochemically determined binding epitope may have limitations. A comprehensive mutational analysis in which all proposed interacting amino acid residues are exchanged for multiple different amino acids is in progress.

## References

[1] Andries K, Verhasselt P, Guillemont J, Göhlmann HW, Neefs JM, Winkler H, Van Gestel J, Timmerman P, Zhu M, Lee E, Williams P, de Chaffoy D, Huitric E, Hoffner S, Cambau E, Truffot-Pernot C, Lounis N, Jarlier V 2005 A diarylquinoline drug active on the ATP synthase of Mycobacterium tuberculosis. Science 307:223–227. doi:10.1126/science.1106753. 15591164

[2] Koul A, Dendouga N, Vergauwen K, Molenberghs B, Vranckx L, Willebrords R, Ristic Z, Lill H, Dorange I, Guillemont J, Bald D, Andries K 2007 Diarylquinolines target subunit c of mycobacterial ATP synthase. Nat Chem Biol 3:323–324. doi:10.1038/nchembio884. 17496888

[3] de Jonge MR, Koymans LH, Guillemont JE, Koul A, Andries K 2007 A computational model of the inhibition of Mycobacterium tuberculosis ATPase by a new drug candidate R207910. Proteins 67:971–980. doi:10.1002/prot.21376. 17387738

[4] Huitric E, Verhasselt P, Koul A, Andries K, Hoffner S, Andersson DI 2010 Rates and mechanisms of resistance development in Mycobacterium tuberculosis to a novel diarylquinoline ATP synthase inhibitor. Antimicrob Agents Chemother 54:1022–1028. doi:10.1128/AAC.01611-09. 20038615 PMC2825986

[5] Haagsma AC, Podasca I, Koul A, Andries K, Guillemont J, Lill H, Bald D 2011 Probing the interaction of the diarylquinoline TMC207 with its target mycobacterial ATP synthase. PLoS One 6:e23575. doi:10.1371/journal.pone.0023575. 21858172 PMC3157398

[6] Segala E, Sougakoff W, Nevejans-Chauffour A, Jarlier V, Petrella S 2012 New mutations in the mycobacterial ATP synthase: new insights into the binding of the diarylquinoline TMC207 to the ATP synthase C-ring structure. Antimicrob Agents Chemother 56:2326–2334. doi:10.1128/AAC.06154-11. 22354303 PMC3346594

[7] Andries K, Villellas C, Coeck N, Thys K, Gevers T, Vranckx L, Lounis N, de Jong BC, Koul A 2014 Acquired resistance of Mycobacterium tuberculosis to bedaquiline. PLoS One 9:e102135. doi:10.1371/journal.pone.0102135. 25010492 PMC4092087

[8] Petrella S, Cambau E, Chauffour A, Andries K, Jarlier V, Sougakoff W 2006 Genetic basis for natural and acquired resistance to the diarylquinoline R207910 in mycobacteria. Antimicrob Agents Chemother 50:2853–2856. doi:10.1128/AAC.00244-06. 16870785 PMC1538646

[9] Preiss L, Langer JD, Yildiz Ö, Eckhardt-Strelau L, Guillemont JE, Koul A, Meier T 2015 Structure of the mycobacterial ATP synthase Fo rotor ring in complex with the anti-TB drug bedaquiline. Sci Adv 1:e1500106. doi:10.1126/sciadv.1500106. 26601184 PMC4640650

[10] Biuković G, Basak S, Manimekalai MSS, Rishikesan S, Roessle M, Dick T, Rao SP, Hunke C, Grüber G 2013 Variations of subunit ε of the Mycobacterium tuberculosis F1Fo ATP synthase and a novel model for mechanism of action of the tuberculosis drug TMC207. Antimicrob Agents Chemother 57:168–176. doi:10.1128/AAC.01039-12. 23089752 PMC3535943

[11] Van Kessel JC, Hatfull GF 2007 Recombineering in Mycobacterium tuberculosis. Nat Methods 4:147–152. doi:10.1038/nmeth996. 17179933

[12] Hotra A, Suter M, Biuković G, Ragunathan P, Kundu S, Dick T, Grüber G 2016 Deletion of a unique loop in the mycobacterial F-ATP synthase γ subunit sheds light on its inhibitory role in ATP hydrolysis-driven H^+^ pumping. FEBS J 283:1947–1961. doi:10.1111/febs.13715. 26996828

[13] Sassetti CM, Boyd DH, Rubin EJ 2003 Genes required for mycobacterial growth defined by high density mutagenesis. Mol Microbiol 48:77–84. doi:10.1046/j.1365-2958.2003.03425.x. 12657046

[14] Moreira W, Ngan GJ, Low JL, Poulsen A, Chia BC, Ang MJ, Yap A, Fulwood J, Lakshmanan U, Lim J, Khoo AY, Flotow H, Hill J, Raju RM, Rubin EJ, Dick T 2015 Target mechanism-based whole-cell screening identifies bortezomib as an inhibitor of caseinolytic protease in mycobacteria. mBio 6:e00253-15. doi:10.1128/mBio.00253-15. 25944857 PMC4436076

[15] Stover CK, De La Cruz VF, Fuerst TR, Burlein JE, Benson LA, Bennett LT, Bansal GP, Young JF, Lee MH, Hatfull GF, Snapper SB, Barletta RG, Jacobs WR, Bloom BR 1991 New use of BCG for recombinant vaccines. Nature 351:456–460. doi:10.1038/351456a0. 1904554

[16] Diacon AH, Pym A, Grobusch M, Patientia R, Rustomjee R, Page-Shipp L, Pistorius C, Krause R, Bogoshi M, Churchyard G, Venter A, Allen J, Palomino JC, Marez TD, van Heeswijk RPG, Lounis N, Meyvisch P, Verbeeck J, Parys W, de Beule K, Andries K, McNeeley DF 2009 The diarylquinoline TMC207 for multidrug-resistant tuberculosis. N Engl J Med 360:2397–2405. doi:10.1056/NEJMoa0808427. 19494215

